# HeteroDualNet: A Dual Convolutional Neural Network With Heterogeneous Layers for Drug-Disease Association Prediction *via* Chou’s Five-Step Rule

**DOI:** 10.3389/fphar.2019.01301

**Published:** 2019-11-08

**Authors:** Ping Xuan, Hui Cui, Tonghui Shen, Nan Sheng, Tiangang Zhang

**Affiliations:** ^1^School of Computer Science and Technology, Heilongjiang University, Harbin, China; ^2^Department of Computer Science and Information Technology, La Trobe University, Bundoora, VIC, Australia; ^3^School of Mathematical Science, Heilongjiang University, Harbin, China

**Keywords:** drug-disease association prediction, multiple kinds of similarities, neighbouring heterogeneous layer, deep learning, dual convolutional neural network

## Abstract

Identifying new treatments for existing drugs can help reduce drug development costs and explore novel indications of drugs. The prediction of associations between drugs and diseases is challenging because their similarities and relations are complicated and non-linear. We propose a HeteroDualNet model to address this issue. Firstly, three types of matrices are extracted to represent intra-drug similarities, intra-disease similarity and drug-disease associations. The intra-drug similarities consider three drug features and a newly introduced drug-related disease correlation. Secondly, an embedding mechanism is proposed to integrate these matrices in a heterogenous drug-disease association layer (hetero-layer). Further, a neighbouring heterogeneous layer (hetero-layer-N) is constructed to incorporate the biological premise that similar drugs can often treat related diseases. Finally, a dual convolutional neural network is built with hetero-layer and hetero-layer-N as two branches to learn from characteristics of drug-disease and the relations of their neighbours simultaneously. HeteroDualNet outperformed the other four methods in comparison over a public dataset of 763 drugs and 681 diseases in terms of Areas Under the Curves of Receiver Operating Characteristics and Precision-Recall, and recall rate at top *k*. Case study of five drugs further proved the capacity of HeteroDualNet in finding reliable disease candidates of drugs as validated by database records or literature. Our findings show that the embedded heterogenous layers of original and neighbouring drug-disease representations in a dual neural network improved the association prediction performance.

## Introduction

The research and development (R&D) processes of new drugs are time-consuming and expensive. Stringent drug testing and approvals are required for an invented new drug to make it to market. For instance, it takes an average of 15 years from preliminary examination of compounds to clinical trials of drug candidates, and finally to drug marketing, while the estimated investment cost is about 800 million dollars (Adams and Brantner, 2006; Tamimi and Ellis, 2009; Pushpakom et al., 2018). However, even in the case of a significant amount of time and capital investment, the R&D of new drugs still faces high failure risks (Li et al., 2016). Meanwhile, the number of new drugs approved by major drug regulatory agencies around the world is decreasing year by year (Grabowski, 2004; Nosengo, 2016). According to the statistics of the US Food and Drug Administration (FDA), the average success rate of new drugs approved from 2003 to 2011 was less than 10% (Padhy and Gupta, 2011; Hay et al., 2014; Pritchard et al., 2017). Therefore, the conventional R&D productivity of new drugs has been stagnant in the last few decades (Paul et al., 2010).

Given the challenges faced by conventional drug R&D techniques, there are significant needs of innovative drug development strategies to increase R&D productivity, which is one of the essential priorities in the pharmaceutical industry. Drug repositioning techniques, or the so-called reuse of existing drugs, have been proved of its advantages over the conventional drug R&D strategies. (Hurle et al., 2013) Drug repositioning is the process to identify new indications for existing drugs and is playing an essential role in the state-of-the-art drug R&D process. Drug repositioning can be applied to drugs which have been approved to market. Because those drugs have passed the procedures of laboratory, pharmacokinetics, toxicology and safety testing, drug developers can use these drugs in clinical trials directly. In this way, drug repositioning skips those procedures and will significantly reduce the time and financial costs in drug development. At the same time, it also reduces the risks of drug development failure. Thus, drug repositioning has attracted great interests in the pharmaceutical industry and research community (Hurle et al., 2013).

Drug repositioning aims to find potential indications for existing drugs (Shim and Liu, 2014; Chen et al., 2016). Computational methods in biology are playing increasingly important roles in the stimulation, development and finding of new drugs (Chou, 2015). To develop useful predictors for biological systems *via* computing models, Chou’s 5-steps (Chou, 2011; Chou, 2019b) are used by recent publications (Chou, 2019a; Awais et al., 2019; Ehsan et al., 2019; Hussain et al., 2019). These steps provide guidance in the development and validation of computerized methods, which include selection of a valid benchmark dataset for training and testing, representation of samples by effective formulation to reflect intrinsic correlations with the target, development of algorithms for prediction, objective performance evaluation by cross-validation, and consideration of public accessibility by web-server.

Several methods have been proposed to predict drug-disease associations. For example, Chiang and Butte proposed a technique based on the internal correlation of networks to predict the potential drug-disease associations (Chiang and Butte, 2009). Sirota et al. developed a prediction method by integrating the common gene expressions of drugs and diseases (Sirota et al., 2011). Besides, Yang and Agarwal et al. proposed to infer the new drug-disease associations by using the phenotypic information on drug side effects (Yang and Agarwal, 2011). Most of these methods are designed for early-stage drugs which have multiple uses and treatment plans. They cannot be used for association prediction when there are no common gene expressions and side effects information between drugs and diseases.

With the increasing amount and variety of drug-related data, recent research has been focusing on integrating multimodality information to investigate the potential uses of drugs. Gottlieb et al. proposed a classification model which used various associations of drug and disease as distinguish signatures. A logistical regression model was then used to predict the indications of drugs (Gottlieb et al., 2011). A kernel-based strategy was proposed to integrate molecular structure, molecular activity, and phenotypic information for drug repositioning (Wang et al., 2013). Heterogenous networks have also been investigated to predict drug indications. Heterogeneous networks are constructed by associating drugs, diseases, targets and genes. The prediction can be achieved by approaches such as network clustering (Wu et al., 2013), priority ranking (Martinez et al., 2015), network topology measurement (Chen et al., 2015), or iteration (Wang et al., 2014b). Given these heterogeneous networks, some other models integrated multiple chemical features such as chemical phenotype of drugs and molecular characteristics of diseases. Then the prediction of new drug indications can be achieved by proteochemometric models (Dakshanamurthy et al., 2012; Yu et al., 2015), statistical (Iwata et al., 2015) or sparse subspace learning (Liang et al., 2017; Xuan et al., 2019) models.

Most of the above existing methods for drug-disease association predictions are shallow models. The associations between drugs and diseases, however, are non-linear and complicated. It is challenging for these shallow models to dig out advanced level while hidden drug-disease relations. Thus, there are great necessities to develop models to learn the deep representations of drug-disease associations for improved drug indication prediction.

In this work, we propose a novel convolutional network with heterogeneous layers and dual branches, referred to as HeteroDualNet, for drug-disease association prediction. Our first unique contribution is the extraction of three types of matrices for the representation and indexing of intra-drug similarity, drug-disease similarity and drug-disease associations. When constructing intra-drug similarity matrices, we consider both regular drug features, including chemical substructures, domains and annotations of target proteins, and a newly introduced feature calculated by drug-related disease correlations. The second contribution is that we construct a new heterogenous drug-disease association layer (hetero-layer) to associate three types of matrices by a proposed embedding mechanism. Further, a drug-disease association layer with neighbouring information (hetero-layer-N) is constructed by the embedding mechanism to reflect the biological premise that similar drugs can often treat related diseases. Finally, HeteroDualNet is built to predict drug-disease associations with hetero-layer and hetero-layer-N as two branches to learn from both original and neighbouring characteristics of drugs and diseases simultaneously. We also investigate the prediction capacity of the proposed model in therapeutic drug indications by case studies of five drugs.

## Materials and Methods

### Dataset

We obtained the data of drugs and diseases from a published work (Wang et al., 2014a). There are 763 drugs, 681 diseases and 3051 known drug-disease associations. The characteristics of each drug include 881 chemical substructures which were initially derived from the chemical fingerprints extracted from the PubChem database (Wang et al., 2009); 1,426 target protein domains from the InterPro database (Mitchell et al., 2015); and 4,447 target protein annotations obtained from the UniProt database (Uniprot, 2010). The similarities among diseases were calculated by (Wang et al., 2010) and provided in the dataset.

### Hypothesis and Framework

**Figure 1:**
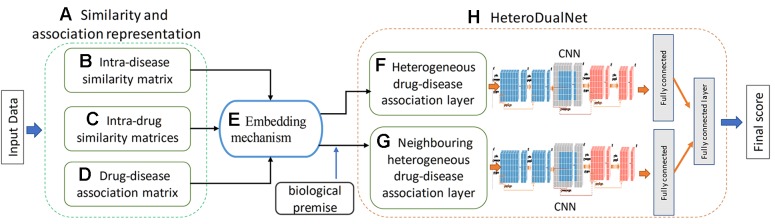
Overview of the proposed HeteroDualNet model for drug-disease association prediction. Given input data, **(A)** similarity and association representations are extracted including **(B)** intra-disease similarity, **(C)** intra-drug similarity, and **(D)** drug-disease association. Then **(E)** an embedding mechanism is proposed to embed these matrices. The final drug-disease association score is obtained by **(H)** HeteroDualNet with **(F)** heterogeneous and biological premise enhanced **(G)** neighboring heterogeneous drug-disease association layers.

We hypothesize that a dual neural network which integrates features of drugs, drug-related disease correlations, and the biological premise of drugs and diseases will improve the performance of drug-disease association predictions. The overview of the proposed method is shown in **Figure 1**. Given the input dataset, the drugs and diseases information is firstly extracted and indexed by three types of similarity matrices in terms of intra-drug, intra-disease and drug-disease. Then, a heterogenous drug-disease association layer, referred by hetero-layer, is constructed by a proposed embedding mechanism to associate those matrices among drugs and diseases. Another heterogeneous layer with neighbouring information, denoted by hetero-layer-N, is built to represent the biological premise that similar drugs can often treat related diseases. Lastly, the dual convolutional neural network is constructed by integrating hetero-layer and hetero-layer-N using a fully connected layer.

### Drug and Disease Similarity and Association Representation

We define three types of matrices to represent and index the information of drugs and diseases in terms of intra-drug similarity, intra-disease similarity and drug-disease associations.

#### Intra-Disease Similarity Matrix

Intra-disease similarities were calculated and provided by (Wang et al., 2010) based on semantic information of diseases (Wang et al., 2010). This information was also used in published work such as Liang et al. (2017) and Zhang et al. (2018). The similarity between disease *d* *_i_* and the disease *d* *_j_* is denoted by ***D***(*i,j*) ∈[0,1]. where is the intra-disease similarity matrix and *N* *^DI^* is the number of diseases. The greater *D*(*i,j*) is, the higher similarity between diseases *d* *_i_* and *d* *_j_*.

#### Intra-Drug Similarity Matrix

Four intra-drug similarity matrices are obtained by calculating the similarities between drugs from four perspectives, including the chemical substructures, target protein domain information, target protein annotations and the related disease information of drugs.

The first three intra-drug similarity matrices of chemical substructure, domain and annotation information of target proteins represent that if two drugs have more common chemical substructures, target protein domains or gene ontology information, the more similar they are. Thus, we calculate these three intra-drug similarity matrices by cosine similarity measurement (Liang et al., 2017).

To calculate the first three intra-drug similarity matrices, we firstly obtain matrices of features and drugs. The feature matrix of chemical feature and all the drugs is denoted by $F_{1}\in {\mathbb{R}}^{N_{1}^{F}\times N^{DR}}$ where $N_{1}^{F}$ is the number of chemical substructure features, and *N* *^DR^* is the number of drugs. Similarly, the feature matrix of protein domain and drugs is $F_{2}\in {\mathbb{R}}^{N_{2}^{F}\times N^{DR}}$ and that of protein annotation and drugs is $F_{3}\in {\mathbb{R}}^{N_{3}^{F}\times N^{DR}}$, where $N_{2}^{F}$ is the number of target protein domain feature and $N_{3}^{F}$ is the number of target protein annotation. Each element of the vectors is 1 or 0 according to whether the drug has such a feature. Given the dataset used in this paper, $N_{1}^{F}=881$, $N_{2}^{F}=1426$ and $N_{3}^{F}=4‚447$. Let ***f*** *_t,i_* be the feature vector of *i-*th drug *r* *_i_* in the *t-*th feature matrix *F* *_t_* (1 ≤ *t* ≤ 3), the similarity ***R*** *_t_*(*i,j*) between drugs *r* *_i_* and *r* *_j_* in terms of feature *t* is calculated by cosine similarity measurement as

(1) $$R_{\text{t}}\left(i,j\right)=\frac{f_{t,i}\cdot f_{t,j}}{\text{||}f_{t,i}\text{|| ||}f_{t,j}\text{||}}.$$

where *R* *_t_*(*i,j*)∈[0,1] and higher values indicate higher similarity between a pair of drugs.

The fourth intra-drug similarities matrix $R_{4}\in {\mathbb{R}}^{N^{DR}\times N^{DR}}$ is obtained based on the idea that if two drugs are associated with similar diseases, the drugs are more likely to be correlated. Given the dataset of diseases ***DI***={*d* *_k_*|*k*∈[1,*N* *^DI^*]} and intra-disease similarity matrix *D* if *i-*th drug *r* *_i_* is associated with a subset of diseases ***DI*** *_m_* ⊂ ***DI***, and drug *r* *_j_* is related to a disease subset ***DI*** *_n_*, the similarity ***R***_4_(*i,j*) between *i-*th and *j-*th drugs can be obtained by calculating the similarity between ***DI*** *_m_* and ***DI*** *_n_* as proposed in our previous work (Xuan et al., 2019) by

(2) $$R_{4}\left(i,j\right)=\frac{\sum_{k=1}^{num\left(DI_{m}\right)}\max\left(D\left(d_{i,k},d_{j,*}\right)\right)+\sum_{k=1}^{num\left(DI_{n}\right)}\max\left(D\left(d_{j,k},d_{i,*}\right)\right)}{num\left(DI_{m}\right)+num\left(DI_{n}\right)}$$

where *num*(*DI* *_m_*) denotes the number of elements in *DI* *_m_*. *d* *_i,k_* represents the *k*th disease related with drug *r* *_i_*, *d* _j,*_ denotes all the related diseases of drug *r* *_j_*, and *max*(*D*(*d* *_i,k_*,*d* _j,*_)) is the maximum similarity between drug $r_{j}^{'}s$ *k*th related disease and all the related diseases of *r* *_j_*. Similarly, *max*(*D*(*d* *_i,k_*,*d* _j,*_)) denotes the maximum similarity between drug $r_{j}^{'}s$ *k*th related disease and all the associated diseases of *r* *_i_*. The final similarity between *r* *_i_* and *r* *_j_* is obtained by the average maximum similarities between diseases in their relevant disease subsets *DI* *_m_* and *DI* *_n_*.

#### Drug-Disease Association Matrix

The drug-disease association matrix is denoted by $A\in {\mathbb{R}}^{N^{DR}\times N^{DI}}$ where an element can be 0 or 1. 1 indicates that a drug and a disease are related, and the association is available; while 0 represents that the relation between a drug and a disease is unknown. Among all the 763 drugs and 681 diseases in the dataset, 3051 drug-disease associations are available. The remaining unknown associations are to be predicted.

### HeteroDualNet Architecture

The sparsity of drug-disease associations makes it challenging to dig out the hidden characteristics and relations between drugs and diseases. We construct HeteroDualNet, a dual convolutional neural network with heterogeneous layers, to predict drug-disease associations. One branch integrates the three matrices of drugs and diseases by a heterogeneous association layer (hetero-layer); the other branch incorporates the neighbouring information in a neighbouring heterogenous layer (hetero-layer-N). The two heterogeneous layers are learnt by passing through convolutional and pooling layers and joint by a connection module. The final association score is obtained by weighted voting of association scores from two branches.

#### Embedding Mechanism for Heterogeneous Drug-Disease Association Matrix

The heterogenous drug-disease association layer is built upon an embedded matrix of afore-extracted matrices. An embedding mechanism is proposed based on the idea that if two drugs are more similar, the more likely they are associated with related diseases, whereas two similar diseases tend to be associated with similar drugs. Given intra-drug matrices ***R*** *_t_*, drug-disease association matrix *A* and intra-disease matrix *D*, the heterogeneous matrix ***X*** *_L_* of drug *r* *_i_*(*i*∈[1,*N* *^DR^*]) and disease *d* *_k_*(*k*∈[1,*N* *^DI^*]) is obtained by the following embedding procedures.

Firstly, row vectors *R* *_t_*(i,*) are combined sequentially as ***X***_L,11_=[***R***_1_(i,*); ***R***_2_(i,*); ***R***_3_(i,*); ***R***_4_(i,*)] where ***R*** *_t_*(i,*) denotes the *i-*th row in an intra-drug similarity matrix *R* *_t_* which records the *t-*th type of similarities between *r* *_i_* and all drugs, *t* = 1,2,3,4 denotes chemical substructures, target protein domains, target protein annotations and related disease information respectively. Secondly, the transposed column vector ***A*** **^T^**(*,k) is concatenated under ***R*** *_4_*(i,*) as ***X*** *_L_*_,21_ where ***A***(*,k) is the *k*th column of *A* which contains the associations between *d* *_k_* and all the drugs. Thirdly, *A*(i,*) is repeated four times and spliced to the right of each row in ***X*** *_L_*_,11_ as ***X*** *_L,_*_12_=[***A***(i,*); ***A***(i,*); ***A***(i,*); ***A***(i,*)] where ***A***(i,*) denotes the *i*th row of *A* which includes the associations between *r* *_i_* and all the diseases. Lastly, ***D***(k,*) is spliced under *X* *_L_*_,12_ where ***D***(k,*) is the *k*th row of ***D*** containing the similarities between *d* *_k_* and all the diseases. The final embedded matrix $X_{L}\in {\mathbb{R}}^{5\times \left(N_{r}+N_{d}\right)}$ of drug *r* *_i_* and disease *d* *_k_* is formed as

(3) $$X_{L}\text{=}\left[\begin{array}{cc} X_{L,11} & X_{L,12}\\ X_{L,21} & X_{L,22} \end{array}\right]\;=\;\left[\begin{array}{cc} \begin{array}{c} \begin{array}{c} \begin{array}{c} \begin{array}{c} R_{1}\left(i,*\right)\\ R_{2}\left(i,*\right) \end{array}\\ R_{3}\left(i,*\right) \end{array}\\ R_{4}\left(i,*\right) \end{array}\\ A^{T}\left(*,k\right) \end{array} & \begin{array}{c} \begin{array}{c} \begin{array}{c} \begin{array}{c} A\left(i,*\right)\\ A\left(i,*\right) \end{array}\\ A\left(i,*\right) \end{array}\\ A\left(i,*\right) \end{array}\\ D\left(k,*\right) \end{array} \end{array}\right]$$

**Figure 2:**
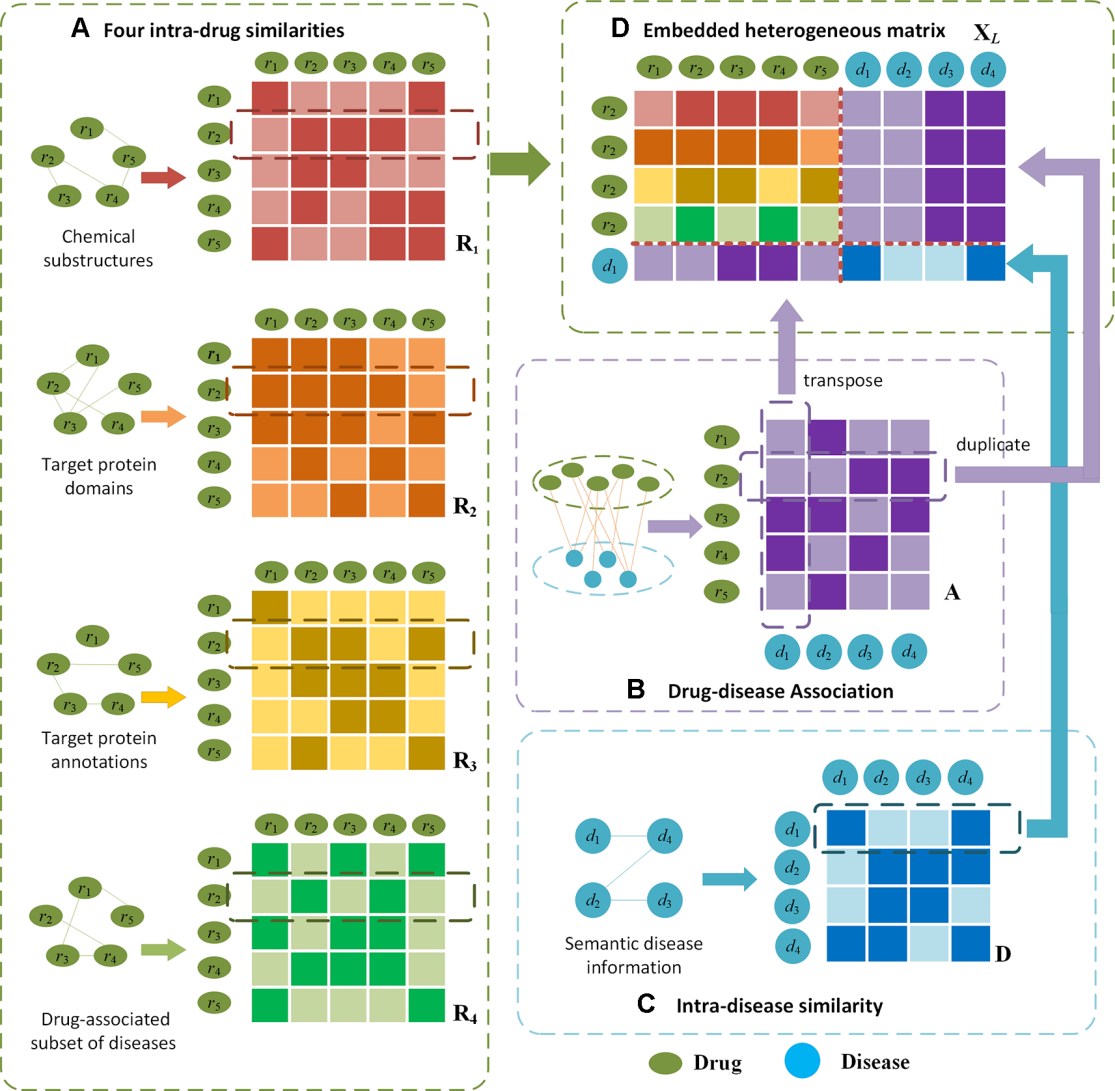
Illustration of the proposed embedding mechanism for heterogenous drug-disease association matrix. Given drug *r*_2_ and disease *d*_1_ as an example, **(D)** the heterogeneous matrix is obtained by integrating **(A)** four types of intra-drug similarities, **(B)** drug-disease associations and **(C)** intra-disease similarities. In **(A)** and **(C)**, darker colours indicate higher similarities; in **(B)** darker colour represents the drug-disease association is available.

Given such a heterogeneous matrix *X* *_L_*, the unknown drug-disease relations can be inferred *via* the correlations between diseases. In the meanwhile, the unavailable associations can be derived upon the similarities between drugs. In **Figure 2**, we illustrate the embedding procedure and use drug *r*_2_ and disease *d*_1_ whose association is unknown as an example. If *r*_2_ is very similar to *r*_3_ and *r*_4_ (as shown in **Figure 2A**),*r*_3_ and *r*_4_ are closely associated with *d*_1_(**Figure 2B**), it can be inferred that *r*_2_ is more likely to be associated with *d* *_1_*. Alternatively, if *d* *_1_* is similar to *d* *_4_* (shown in **Figure 2C**), and *d* *_4_* is related with *r*_2_ (**Figure 2B**), a high possibility that *r*_2_ is associated with *d* *_1_* can be derived.

#### Neighbouring Heterogeneous Association Matrix

The neighbouring heterogeneous drug-disease association matrix *X* *_L–N_* embeds the neighbours of drug *r* *_i_* and disease *d* *_k_*. The embedding is proposed based on the biological premise that if the neighbours of a drug are associated with the neighbours of a disease, there is a high probability that the drug and the disease are associated. The embedding procedures considering the neighbours of *r* *_i_* and *d* *_k_* is: Firstly, we find drugs *r* *_m_*, *r* *_n_*, *r* *_p_*, and *r* *_q_* which are the most similar neighbours of drug *r* *_i_* in ***R***_1_, ***R***_2_, ***R***_3_ and ***R***_4_ respectively. We also find *d* *_l_*, the most similar neighbour of *d* *_k_*, in *D*. Similar with ***X*** *_L,_*_11_, the *m-*th row of ***R***_1_, *n*th row of ***R***_2_, *p-*th row of ***R***_3_, and *q*th row of ***R***_4_ are combined from top to bottom to form ***X*** *_L_*_–_ *_N,_*_11_. Secondly, the *l-*th column of ***A*** indicating the association between the most similar disease *d* *_l_* and all the drugs is transposed and concatenated under ***X*** *_L_*_–_ *_N,_*_11_ as ***X*** *_L_*_–_ *_N,_*_21_. Thirdly, row vectors ***A***(*m*,*), ***A***(*n*,*), ***A***(*p*,*), ***A***(*q*,*) are spliced to the right of each row in ***X*** *_L_*_–_ *_N,_*_11_, where ***A***(*m*,*), ***A***(*n*,*), ***A***(*p*,*), ***A***(*q*,*) indicate the associations between drugs *r* *_m_*,*r* *_n_*,*r* *_p_* and *r* *_q_* and all the diseases. Lastly, the *l-*th row of *D* containing the similarities between disease *d* *_l_* and all the other diseases is concatenated under *X* *_L_*_–_ *_N,_*_21_. In such a way, the final embedding of most similar neighbours of *r* *_i_* and *d* *_k_* is formed as $X_{L-N}\in {\mathbb{R}}^{5\times \left(N^{DR}+N^{DI}\right)}$:

(4) $$X_{L}\text{=}\left[\begin{array}{cc} X_{L-N,11} & X_{L-N,12}\\ X_{L-N,21} & X_{L-N,22} \end{array}\right]\;=\;\left[\begin{array}{cc} \begin{array}{c} \begin{array}{c} \begin{array}{c} \begin{array}{c} R_{1}\left(m,*\right)\\ R_{2}\left(n,*\right) \end{array}\\ R_{3}\left(p,*\right) \end{array}\\ R_{4}\left(q,*\right) \end{array}\\ A^{T}\left(*,l\right) \end{array} & \begin{array}{c} \begin{array}{c} \begin{array}{c} \begin{array}{c} A\left(l,*\right)\\ A\left(l,*\right) \end{array}\\ A\left(l,*\right) \end{array}\\ A\left(l,*\right) \end{array}\\ D\left(l,*\right) \end{array} \end{array}\right]$$

**Figure 3:**
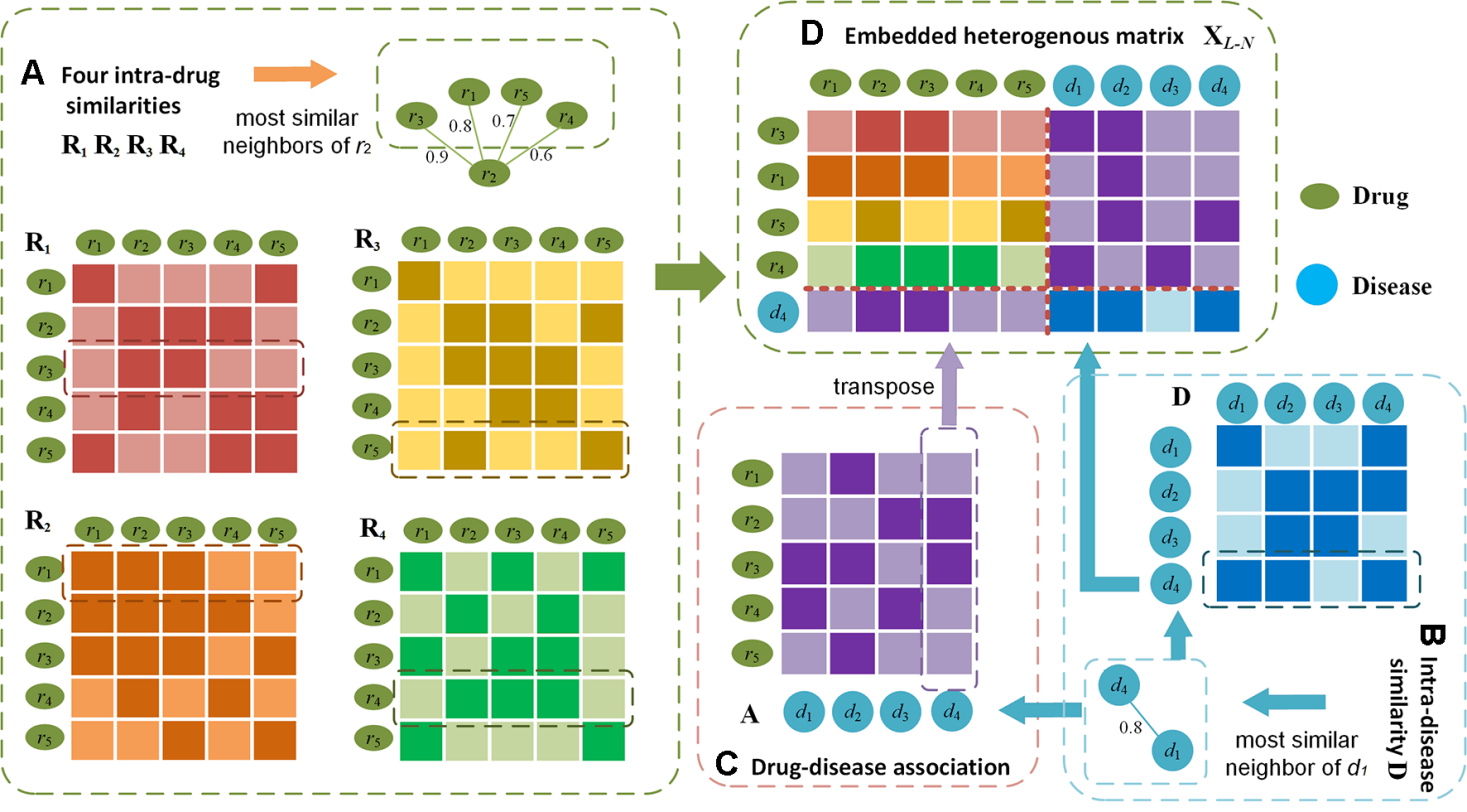
Illustration of the embedding procedure for neighbouring heterogeneous matrix. Using drug *r*_2_ and disease *d*_1_ as an example, **(D)** the final matrix is obtained by finding the most similar neighbours (e.g. *r*_3_,*r*_1_,*r*_5_,*r*_4_) of *r*_2_ calculated from **(A)** four intra-drug similarities respectively, the most similar neighbour (e.g. *d*_4_) of drug *d*_1_ by **(B)** intra-drug similarity matrix, and **(C)** drug-disease associations. In **(A)** and **(B)**, darker colours indicate higher similarities; in **(C)** darker colour represents the drug-disease association is available.

In *X* *_L_*_–_ *_N_*, the most similar neighbours of drugs and diseases serve as the bridge to propagate associations. In **Figure 3**, we use drug *r*_2_ and disease *d*_1_ whose association is unknown as an example to illustrate the embedding procedure and information propagations. For instance, assume we find that drug *r*_2_ likes *r*_3_ the most in ***R***_1_, *r*_1_ in ***R***_2_,*r*_5_ in ***R***_3_, and *r*_4_ in ***R***_4_(**Figure 3A**), and *d*_1_ likes *d*_4_ the most in *D* (as shown in **Figure 3B**). In the embedded matrix ***X*** *_L–N_*, the left part indicates that all $r_{2}^{'}s$ most similar neighbours (*r*_3,_ *r*_1,_ *r*_5,_ *r*_4_) are very similar to *r*_2_ and *r*_3_. Because *d*_4_ is associated with bridging drugs *r*_2_ and *r*_3_ based on *A* (**Figure 3C**), it can be inferred that there is a high probability that *r*_2_ and *d*_1_ are associated. The right part shows that the majority of $r_{2}^{'}s$ most similar neighbours are related with *d*_2_. As $d_{1}^{'}s$ most similar neighbour *d*_4_ is closely related to the bridging disease *d*_2_ by ***D***, it can be derived that *d*_1_ is probably related with *r*_2_.

#### HeteroDualNet for Association Prediction

**Figure 4:**
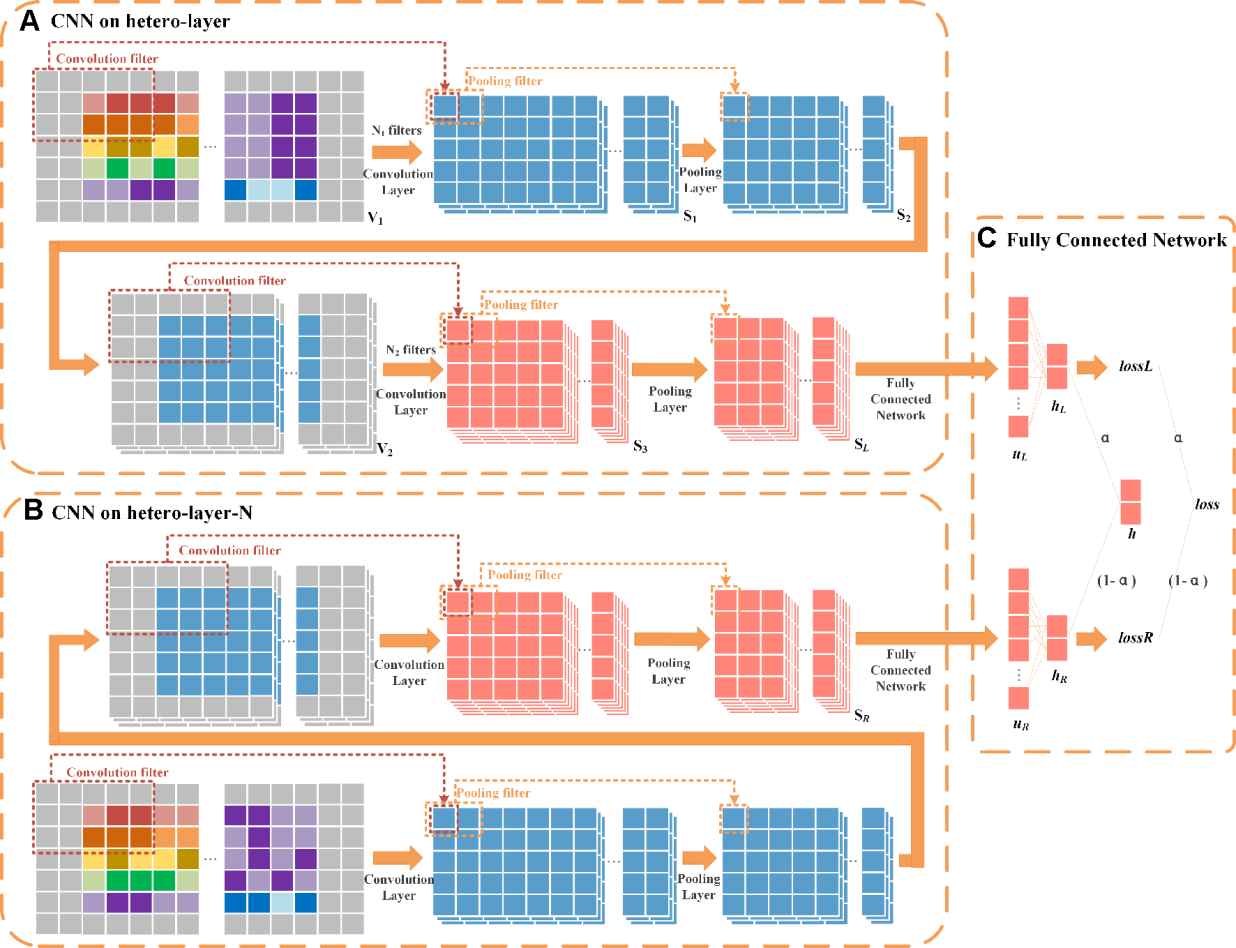
Schematic diagram of HeteroDualNet. **(A)** One branch over hetero-layer of drug-disease characteristics and **(B)** one branch over the neighbouring heterogeneous layer (hetero-layer-N) are connected by **(C)** an integration module for final association score prediction. Three 3×5 filters in 1^st^ convolution, six 3×5 filters in 2^nd^ convolution, a sliding window of 1 × 2 in 1^st^ and 2^nd^ pooling are used for illustration.

The architecture of HeterDualNet is given in **Figure 4**. The hetero-layer and hetero-layer-N are obtained by zero padding heterogenous matrices ***X*** *_L_* and ***X*** *_L–N_*. One branch in the dual CNN model alternates two convolution and two pooling operations over hetero-layer (**Figure 4A**), the other branch is built where hetero-layer-N is convolved and pooled for neighbouring feature representations (**Figure 4B**). These two branches are connected by a fully connected network to achieve the final association score between *r* *_i_* and *d* *_k_* (**Figure 4C**). Same network settings are used in the two branches, thus we introduce the branch with hetero-layer in detail.

**Convolutional module on hetero-layer.** The heterogeneous matrix ***X*** *_L_* is firstly padded with zeros to preserve the marginal information of matrices. In the first convolutional layer, we set *N* *_1_* filters where each filter is with width and length of $n_{w_{c1}}$ and $n_{l_{c1}}$. The hetero-layer is thus denoted as $V_{1}\in {\mathbb{R}}^{\left(5\text{+}2l\right)\times \left(N_{r}+N_{d}+2p\right)}$ where $l\text{ = }\left(n_{w_{c1}}-1\right)/2$ $p\text{ = }\left(n_{l_{c1}}-1\right)/2$. The case when *N*_1_=3, $n_{w_{c1}}=3$, and $n_{l_{c1}}=5$ is illustrated as an example in **Figure 4A**. The weight parameter matrix of a *n-*th filter in the first convolutional layer is denoted by $W_{1,n}\in {\mathbb{R}}^{n_{w_{c1}}\times n_{l_{c1}}}$, *n*∈[1,*N*_1_]. The step size of a sliding window is set to be 1×1. The output of the first convolutional layer is obtained as $S_{1}\in {\mathbb{R}}^{N_{1}\times 5\times \left(N_{r}+N_{d}\right)}$ where $S_{1,n}\in {\mathbb{R}}^{5\times \left(N_{r}+N_{d}\right)}$ is the *n*-th output after *V* *_1_* is scanned by the *n*-th filter as

(5) $$S_{1,n}=\left[\begin{array}{cccc} S_{1,n\left(1,1\right)} & S_{1,n\left(1,2\right)} & \cdots & S_{1,n\left(1,N_{r}+N_{d}\right)}\\ S_{1,n\left(2,1\right)} & S_{1,n\left(2,2\right)} & \cdots & S_{1,n\left(2,N_{r}+N_{d}\right)}\\ \vdots & \vdots & \ddots & \vdots \\ S_{1,n\left(5,1\right)} & S_{1,n\left(5,2\right)} & \cdots & S_{1,n\left(5,N_{r}+N_{d}\right)} \end{array}\right]$$

where *S*_l,_ *_n_*_(_ *_i,j_*_)_ is the element in the *i*-th row and the *j*-th column of *S*_l,_ *_n_* as:

(6) $$S_{1,n\left(i,j\right)}=g\left(V_{1}^{'}\cdot W_{1,n}+b_{1,n}\right)$$

where *b*_l,_ *_n_* is the bias, “g” denotes the dot product, and *g* is a ReLu function. *V*_l(_ *_i,j_*_)_ is the element in the *i*-th row and the *j*-th column of *V*_1_. When the filter slides to the position where *V*_1(_ *_i,j_*_)_ is the center point, |$V_{1\left(i,j\right)}^{'}\in {\mathbb{R}}^{n_{w_{c1}}\times n_{l_{c1}}}$| is formed by all the elements in the filter window as follow

(7) $$V_{1\left(i,j\right)}^{'}=\left[\begin{array}{ccccc} V_{1\left(i-1,j-2\right)} & V_{1\left(i-1,j-1\right)} & V_{1\left(i-1,j\right)} & V_{1\left(i-1,j+1\right)} & V_{1\left(i-1,j+2\right)}\\ V_{1\left(i,j-2\right)} & V_{1\left(i,j-1\right)} & V_{1\left(i,j\right)} & V_{1\left(i,j+1\right)} & V_{1\left(i,j+2\right)}\\ V_{1\left(i+1,j-2\right)} & V_{1\left(i+1,j-1\right)} & V_{1\left(i+1,j\right)} & V_{1\left(i+1,j+1\right)} & V_{1\left(i+1,j+2\right)} \end{array}\right]$$

We set the width and length of the sliding window in the first pooling layer as $n_{w_{p1}}$ and $n_{l_{p1}}$ ($n_{w_{p1}}=1$ and $n_{l_{p1}}=2$ as an example in **Figure 4**) and the step size as. The output of the first pooling $S_{2}\in {\mathbb{R}}^{N_{1}\times 5\times \left(N_{r}+N_{d}\right)/2}$. is obtained by a max-pooling operation where the *n*-th output $S_{2,n}\in {\mathbb{R}}^{5\times \left(N_{r}+N_{d}\right)/2}$ is

(8) $$S_{2,n}=\left[\begin{array}{cccc} S_{2,n\left(1,1\right)} & S_{2,n\left(1,2\right)} & \cdots & S_{2,n\left(1,\left(N_{r}+N_{d}\right)/2\right)}\\ S_{2,n\left(2,1\right)} & S_{2,n\left(2,2\right)} & \cdots & S_{2,n\left(2,\left(N_{r}+N_{d}\right)/2\right)}\\ \vdots & \vdots & \ddots & \vdots \\ S_{2,n\left(5,1\right)} & S_{2,n\left(5,2\right)} & \cdots & S_{2,n\left(5,\left(N_{r}+N_{d}\right)/2\right)} \end{array}\right]$$

where $S_{2,n\left(i,j\right)}$ is the maximum value between $S_{1,n\left(i,2j\text{-}1\right)}$ and $S_{1,n\left(i,2j\right)}$ defined as

(9) $$S_{2,n\left(i,j\right)}=max\left(S_{1,n\left(i,2j-1\right)},S_{1,n\left(i,2j\right)}\right)$$

By padding *S*_2,_ *_n_* with zeros, *V*_2_ is obtained as $V_{2}\in {\mathbb{R}}^{\left(5\text{+}2l\right)\times \left(N_{r}+N_{d}+2p\right)}$ where $l=\left(n_{w_{c2}}-1\right)/2$ and $p=\left(n_{l_{c2}}-1\right)/2$. The number of filters is set as *N*_2_ in the second convolution. The output of the second convolution is obtained as $S_{3}\in {\mathbb{R}}^{N_{2}\times 5\times \left(N_{r}+N_{d}\right)/2}$. In the second pooling layer, we set the width and length of the sliding window as $n_{w_{p2}}$ and $n_{l_{p2}}$, and the step size as $n_{w_{p2}}\times n_{l_{p2}}$. For instance, the case when *N*_2_ = 6,$n_{w_{p2}}=\;1$ and $n_{l_{p2}}=\;2$ is illustrated as an example in **Figure 4**. The output of the second pooling is obtained as $S_{4}\in {\mathbb{R}}^{N_{2}\times 5\times \left(N_{r}+N_{d}\right)/4}$ which is also the final output. Let ***S*** *_L_* represent the final output of this branch, ***S*** *_L_* = ***S***_4_.

**Convolutional module on hetero-layer-N.** The settings of convolution and pooling operations on hetero-layer-N is the same as the above branch. Let *S* *_R_* denote the final output given ***X*** *_L_*_–_ *_N_* as inputs, $S_{R}\in {\mathbb{R}}^{N_{2}\times 5\times \left(N_{r}+N_{d}\right)/4}$.

**Final integration module**. The integration of two branches is obtained by firstly flattening ***S*** *_L_* and ***S*** *_R_* as vectors $u_{L},u_{R}\in {\mathbb{R}}^{1\times \left(N_{2}\times 5\times \left(N_{r}+N_{d}\right)/4\right)}$. *u* *_L_* and *u* *_R_* are then fed into a fully connected layer (as shown in **Figure 4C**).

The association score $h_{L}\in {\mathbb{R}}^{2\times 1}$ between drug *r* *_i_* *r* *_i_* and disease *d* *_k_* in one branch is obtained as

(10) $$h_{L}=softmax\left(W_{L}u_{L}^{T}+b_{L}\right)$$

where $W_{L}\in {\mathbb{R}}^{2\times \left(5\times \left(N_{r}+N_{d}\right)/4\times n_{2}\right)}$ is the weight parameter matrix, and *b* *_L_* is a bias vector. *h* *_L_*(1) contains the probability that *r* *_i_* is associated with *d* *_k_* and *h* *_L_*(2) is the probability that *r* *_i_* and *d* *_k_* are not associated. Similarly, the association score *h* *_R_* of the other branch is calculated by

(11) $$h_{R}=softmax\left(W_{R}u_{R}^{T}+b_{R}\right)$$

The final association score ***h*** is obtained by a weighted fusion of ***h*** *_L_* and ***h*** *_R_* as

(12) $$h=\alpha h_{L}+\left(1-\alpha \right)h_{R},\;s.t.\;0\leq \alpha \leq 1$$

where *α* is a regulation parameter to balance the contributions of two branches. Let *lossL* and *lossR* denote the losses of two branches as:

(13) $$lossL=\min{\left\|h_{L}-y\right\|}_{F}^{2},\;lossR=\min{\left\|h_{R}-y\right\|}_{F}^{2}$$

where $y=\left[\begin{array}{c} y_{0}\\ y_{1} \end{array}\right]$ is the probability that drug *r* *_i_* and disease *d* *_k_* are associated. If *r* *_i_* and *d* *_k_* are associated, *y* *_0_*=0 and *y*_1_=1, otherwise *y*_0_=1 and *y*_1_=0. The final loss *loss* is obtained by

(14) $$loss=min\;\alpha {\left\|h_{L}-y\right\|}_{F}^{2}+\left(1-\alpha \right){\left\|h_{R}-y\right\|}_{F}^{2}$$

where the regulation parameter *α* is the same as that in Equation 12. With the network architecture and loss function, the parameters are randomly initialized and adjusted in the training process until the loss function is minimized. Given three types of drug-disease matrices, the final drug-disease association score can be predicted by the trained HeteroDualNet model.

In order to reduce the impact of overfitting which is caused by the number of parameters in the proposed model based on dual CNN, we adopt the widely used dropout strategy to prevent the overfitting of HeteroDualNet. During each iteration process for training the model, HeteroDualNet randomly ignores some neurons to ensure that the trained model will have a good generalization ability.

## Experimental Evaluations and Discussions

### Experimental Setup

The drug-disease samples with known associations are regarded as one class (*L*_1_), while those pairs with unknown associations are considered as the other class (*L*_2_). In total, there are 3051 *L*_1_ samples, and 763*681-3051 = 516552 *L*_2_ samples. Because *L*_1_ and *L*_2_ samples are largely imbalanced, undersampling strategy is used to address this issue. We divided the data into two subsets. One subset A is composed of 3051 *L*_1_ samples and 3051 *L*_2_ samples, while the second subset B contains the remaining 516552 – 3051 *L*_2_ samples.

Five-fold cross-validation is performed to evaluate the prediction performance of HeteroDualNet and other compared models. The same training and testing data are used for the training and testing of the models. In each round of validation, the samples in subset A are equally divided into five parts where four parts are used as the training dataset, and one part together with subset B are used for testing.

As the calculation of the *4*-th intra-drug similarities matrix ***R***_4_ involves drug-disease association matrix ***A*** and intra-disease matrix ***D*** to ensure that there is no testing data information in the training dataset, ***R***_4_ is recalculated by removing drug-disease samples that appear in training in each round of validation.

### Comparison Methods and Evaluation Metrics

To evaluated the contributions of the proposed HeteroDualNet architecture and heterogenous drug-disease similarity representations, our model is compared with other four prediction methods including TL_HGBI (Wang et al., 2014b), MBiRW (Luo et al., 2016), LRSSL (Liang et al., 2017), and SCMFDD (Zhang et al., 2018). LRSSL is based on three drug features without considering neighbouring information and our proposed fourth intra-drug similarity from drug-related disease correlations. MBiRW used only one type of drug feature. SCMFDD and TL_HGBI used matrix decomposition and heterogeneous networks, but they didn’t consider neighbouring information and multiple features.

The prediction performance is comprehensively evaluated by true positive rate (*TPR*), false positive rate (*FPR*), the Receiver Operating Characteristic (ROC) area under curve (ROC AUC), the Precision-Recall area under curve (PR AUC) and recall rate under different top *k* values. TPR and FPR are calculated as

(15) $$TPR=\frac{TP}{TP+FN},\;FPR=\frac{FP}{TN+FP},$$

where *TP* (*FN)* is the number of positive samples that are correctly identified (misidentified), *TN* (*FP)* is the number of correctly identified (misidentified) negative samples. A sample is regarded as a positive sample when its predicted association score is greater than a threshold *θ*. If the testing sample’s score is smaller than *θ*, it is identified as a negative sample. The values of *FPR* and *TPR* are calculated by setting different values of *θ*. The average ROC AUC value of all the evaluated drugs is used as the overall prediction performance of a method.

Since two classes are heavily imbalanced, the evaluation by PR AUC is more appropriate than ROC AUC in our study. Thus, PR AUC is also compared among different methods. *Precision* and *Recall* are defined by

(16) $$Precision=\frac{TP}{TP+FP},\;Recall=\frac{TP}{TP+FN}$$

where *Precision* represents the ratio between the number of correctly identified positive samples and all samples which are predicted to be positive samples, and *Recall* represents the ratio of the correctly identified positive samples to all the positive samples. Meanwhile, because the top-ranked results are of greater interest in real practices, which are often considered by biologists for further validation, we also calculate the recall rate in top *k* ranked results. The higher the recall rate for the top *k* disease, the more drug-related diseases can be predicted by the model.

### Experimental Results and Discussion

**Figure 5:**
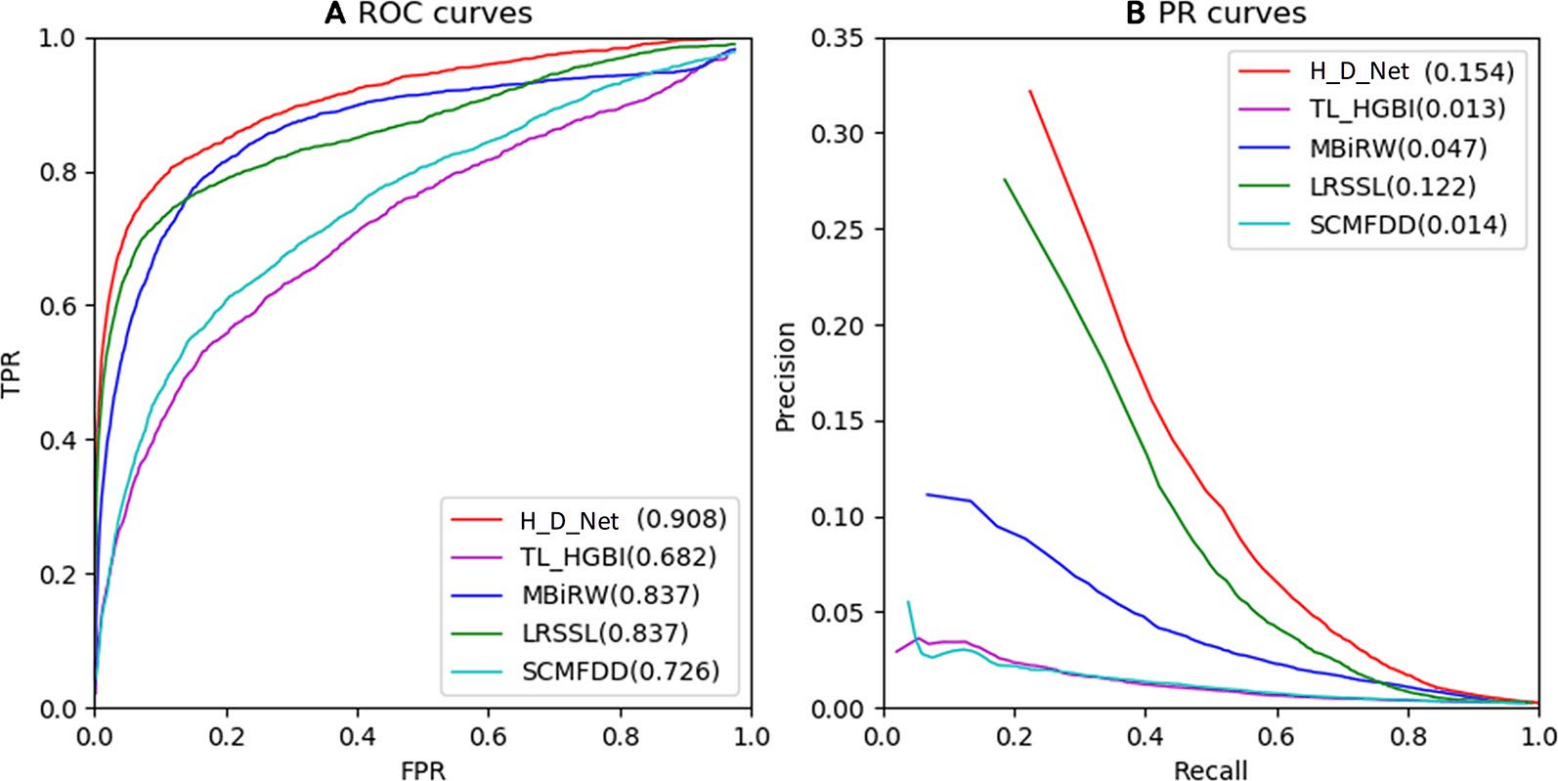
Comparison between the proposed HeteroDudalNet model (H_D_Net) against four other methods by Receiver Operating Characteristic (ROC) **(A)** and Precision-Recall (PR) **(B)** curves.

The ROC and PR of all the methods using all the 763 drugs are shown in **Figure 5**. The AUC results are given in **Table 1**. As shown by **Figure 5A** and **Table 1**, our model achieved the highest AUC of 0.908 among all the methods in comparison, which is 7.1% greater than the second best MBiRW model, 18.2% higher than the SCMFDD method, and 22.6% higher than the TL_HGBI method. As shown by **Figure 5B** and **Table 1**, HeteroDudalNet achieved the best performance where PR AUC reached 0.154, which was 3.2%, 10.7%, 14%, and 14.1% better than the that of LRSSL, MBiRW, SCMFDD and TL_HGBI models respectively.

**Table 1:** Receiver Operating Characteristic area under curve (ROC AUC) and Precision-Recall area under curve (PR AUC) of all the methods in comparison.

	Average performance on 763 drugs				
	HeteroDualNet	TL_HGBI	MBiRW	LRSSL	SCMFDD
ROC AUC	0.908	0.723	0.855	0.845	0.611
PR AUC	0.154	0.031	0.045	0.089	0.006

As shown by the ROC and PR evaluation results, HeteroDudalNet outperformed the second best LRSSL because of the integration of neighbouring information on drugs and diseases and the intra-drug similarity calculated by correlations of drug-related diseases. Compared with LRSSL which considered three types of drug features, the third best model MBiRW considered only one type of drug feature in an adopted a random walk-based model, which resulted in a much lower prediction score. Without considering neighbouring associations and multiple features, SCMFDD and TL_HGBI methods failed to achieve satisfactory prediction performance although they used matrix decomposition and heterogeneous networks.

**Figure 6:**
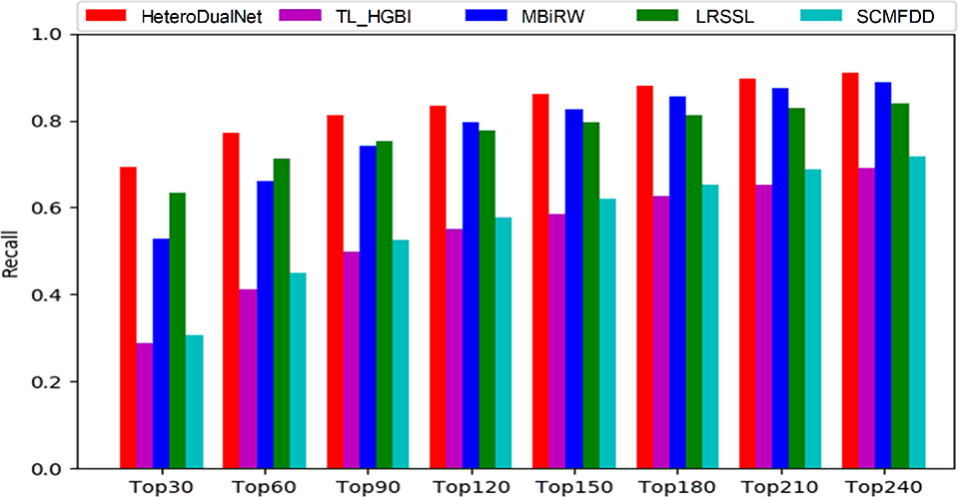
The recalls across all the tested drugs at different top *k* cutoffs.

The average performance over all the 763 drugs in terms of recall rate given different top *k* values is shown in **Figure 6**. The higher the recall rate for the top *k* diseases, the more drug-related diseases can be predicted by a computing model. When increasing the value of *k* from 30 to 240 with a step of 30, the average recall rate of our method is the best among all the models in comparison. When examining the top 30, 60 and 90 diseases, our model achieved recall rates of 69.2%, 77%, and 83.5%, and the second best was obtained by LRSSL with recall rates of 63.4%, 71.3%, and 77.7% respectively. The third-ranked model MBiRW performed slightly worse than LRSSL where the results were 52.9%, 66% and 74.2%. When *k* was increased from 90 to 240, MBiRW started to perform better than LRSSL and achieved its highest recall rate of 88.7% when k was 240, while our model obtained the best rate of 90.9% among all the methods. Overall, the top *k* recall rates of SCMFDD and TL_HGBI were significantly lower than the other techniques in comparison.

As shown by the top *k* recall rate test, our model achieved the best performance, which could be useful for biologists to conduct clinical experiments because the highest ranked list contains more real drug-disease associations. As shown by the results when *k* was smaller than 90, our model and LRSSL outperformed the other methods because of the consideration of multiple drug features. The comprehensive representation of drugs concerning similarities in various perspectives contributes to digging out the potential associations between drugs and diseases. When *k* > 90, the number of common features between drug and disease may be decreasing when compared with smaller *k* values. Thus, considering only multiple features could not always guarantee a good prediction result. MBiRW performed better than LRSSL due to the consideration of global information in a random walk based network. By incorporating three drug characteristics, the calculated correlations between drug-related diseases as intra-drug similarities, and neighbouring information of similar drugs and diseases, our model achieved better results than LRSSL and MBiRW.

### Case Studies of Five Drugs

To further evaluate and demonstrate the effectiveness of the proposed HeteroDualNet in finding reliable disease candidates of drugs, we conducted case studies of five drugs, including ciprofloxacin, ceftriaxone, ofloxacin, ampicillin and cefotaxime. Two public drug disease databases, Comparative Toxicogenomics Database (CTD) and DrugBank, were used to verify and confirm the predicted drug-disease associations by the proposed model. CTD is funded by the National Institute of Environmental Health Sciences which contains information of drugs and drugs’ effects on diseases extracted from published literature. DrugBank is supported by the Health Research Institute of Canada, the Alberta Innovation-Health Solutions and Metabolic Innovation Center. Drugs’ clinical trial information can be found in DrugBank, which includes drugs and diseases in experiments.

For each of the five drugs, we ranked the predicted diseases according to the relevance scores in descending order. The top 10 ranked diseases are used for verification and listed in **Table 2**. Among all the 50 diseases, 31 disease-drug association information can be found in CTD, and 17 association information can be found in the DrugBank as shown in **Table 2**. The results demonstrated that the predicted candidate diseases are indeed associated with the corresponding drugs. Also, in the CTD database, the association between Ciprofloxacin and Eye Infections, Bacterial can be found in the literature. For the two diseases which cannot be found in CTD and DrugBank, one of them can be verified by ClinicalTrials.gov (https://clinicaltrials.gov/) which records a wealth of clinical research information on various drugs and related diseases by National Institutes of Health (NIH) and the Food and Drug Administration (FDA). Therefore, there is only one disease candidate of drug ampicillin, which is Pseudomonas Infections, cannot be proved by the three databases and is labelled as unconfirmed in **Table 2**. The case studies demonstrated that our model can be used as an effective tool to predict the relations between drugs and diseases. At the same time, it has the capacity to provide computer-aided guidance for biologists in clinical trials.

**Table 2:** Top 10 related candidate diseases of ciprofloxacin, ceftriaxone, ofloxacin, ampicillin and cefotaxime.

Drug name	Rank	Disease name	Description	Rank	Disease	Description
ciprofloxacn	1	Pneumonia, Bacterial	CTD	6	Gram-Positive Bacterial Infections	CTD
	2	Salmonella Infections	CTD	7	Eye Infections, Bacterial	Literature (Marino et al., 2013)
	3	Bacterial Infections	CTD	8	Soft Tissue Infections	CTD
	4	Streptococcal Infections	DrugBank	9	Enterobacteriaceae Infections	CTD
	5	Gram-Negative Bacterial Infections	CTD	10	Helicobacter Infections	CTD
ceftriaxone	1	Gram-Negative Bacterial Infections	CTD	6	Haemophilus Infections	CTD
	2	Bacterial Infections	CTD, ClinicalTrials	7	Gram-Positive Bacterial Infections	CTD
	3	Septicemia	DrugBank	8	Skin Diseases, Infectious	DrugBank
	4	Respiratory Tract Infections	CTD	9	Wound Infection	ClinicalTrials
	5	Pseudomonas Infections	DrugBank	10	Eye Infections, Bacterial	DrugBank
ofloxacin	1	Eye Infections, Bacterial	ClinicalTrials, DrugBank	6	Pseudomonas Infections	CTD
	2	Gram-Negative Bacterial Infections	DrugBank	7	Bacterial Infections	CTD
	3	Sinusitis	CTD	8	Bacteroides Infections	DrugBank
	4	Streptococcal Infections	CTD	9	Gram-Positive Bacterial Infections	CTD
	5	Pneumonia, Bacterial	CTD	10	Enterobacteriaceae Infections	DrugBank
ampicillin	1	Pseudomonas Infections	unconfirmed	6	Proteus Infections	CTD
	2	Bacterial Infections	CTD	7	Septicemia	DrugBank
	3	Gram-Positive Bacterial Infections	CTD	8	Streptococcal Infections	CTD
	4	Gram-Negative Bacterial Infections	CTD	9	Wound Infection	CTD
	5	Pneumonia, Bacterial	CTD, ClinicalTrials	10	Enterobacteriaceae Infections	DrugBank
cefotaxime	1	Respiratory Tract Infections	CTD, ClinicalTrials	6	Enterobacteriaceae Infections	DrugBank
	2	Pseudomonas Infections	DrugBank	7	Gram-Positive Bacterial Infections	CTD, DrugBank
	3	Gram-Negative Bacterial Infections	CTD, DrugBank	8	Wound Infection	DrugBank
	4	Septicemia	DrugBank	9	Skin Diseases, Infectious	ClinicalTrials
	5	Bacterial Infections	CTD, ClinicalTrials	10	Osteomyelitis	CTD, ClinicalTrials

(1) CTD refers to the Comparative Toxicogenomics Database (CTD), which contains a manually managed drug-disease association. (2) DrugBank refers to the drug-disease association held in the DrugBank database, which collects experimental information of the drug. (3) ClinicalTrials means that the association of drugs with the disease is recorded in the online database ClinicalTrials.gov. (4) literature refers to the literature supporting the association of drugs with the disease. (5) unconfirmed means that there is no evidence that the drug is associated with the disease.

The future direction for developing userful and powerful computerized prediction methods include establishing web-servers to enable public assessibility (Cheng et al., 2017; Cheng et al., 2018; Xiao et al., 2019; Chou, 2019a; Chou, 2019b). Our future work include providing a web-server for the proposed model to increase the impact of computational model in bioinformatics, medical science and medicinal chemistry.

## Conclusion

We present a novel HeteroDualNet model for drug-disease association prediction. Our model incorporates three kinds of drug features, a newly introduced intra-drug similarity based on correlations of drug-related diseases, and neighbouring information of drugs and diseases by constructing embedded drug-disease heterogenous matrices and dual branches in a deep neural network. The evaluation of public dataset and comparison with other four published models demonstrated the improved prediction performance in terms of ROC AUC, PR AUC, and recall rate at top *k*. Case studies of five drugs further proved the effectiveness of our model in finding potential relevant diseases of drugs as validated by database records or literature. Our model can be used as an effective tool to predict the associations between drugs and diseases and provide computer-aided guidance for biologists in clinical trials.

## Data Availability Statement

The datasets generated and analyzed for this study can be found at https://github.com/LiangXujun/LRSSL. 

## Author Contributions

PX, HC, and TS conceived the prediction method, and they wrote the paper. NS and TS developed computer programs. PX, TZ, and TS analyzed the results, and PX, HC, and NS revised the paper.

## Funding

The work was supported by the Natural Science Foundation of China (61972135), the Natural Science Foundation of Heilongjiang Province (LH2019F049, LH2019A029), the China Postdoctoral Science Foundation (2019M650069), the Heilongjiang Postdoctoral Scientific Research Staring Foundation (BHL-Q18104), the Fundamental Research Foundation of Universities in Heilongjiang Province for Technology Innovation (KJCX201805), and the Fundamental Research Foundation of Universities in Heilongjiang Province for Youth Innovation Team (RCYJTD201805).

## Conflict of Interest

The authors declare that the research was conducted in the absence of any commercial or financial relationships that could be construed as a potential conflict of interest.
